# Handheld Device Adapted to Smartphone Cameras for the Measurement of Sodium Ion Concentrations at Saliva-Relevant Levels via Fluorescence

**DOI:** 10.3390/bioengineering2020122

**Published:** 2015-06-02

**Authors:** Michelle Lipowicz, Antonio Garcia

**Affiliations:** School of Biological and Health Systems Engineering, Ira A. Fulton Schools of Engineering, Arizona State University, Tempe, AZ 85287, USA; E-Mail: mlipowic@asu.edu

**Keywords:** smartphone, saliva, sodium ions, fluorescence, oblate spheroid, non-axially-symmetric focusing, low-resource settings

## Abstract

The use of saliva sampling as a minimally-invasive means for drug testing and monitoring physiology is a subject of great interest to researchers and clinicians. This study describes a new optical method based on non-axially symmetric focusing of light using an oblate spheroid sample chamber. The device is simple, lightweight, low cost and is easily attached to several different brands/models of smartphones (Apple, Samsung, HTC and Nokia) for the measurement of sodium ion levels at physiologically-relevant saliva concentrations. The sample and fluorescent reagent solutions are placed in a specially-designed, lightweight device that excludes ambient light and concentrates 470-nm excitation light, from a low-power photodiode, within the sample through non-axially-symmetric refraction. The study found that smartphone cameras and post-image processing quantitated sodium ion concentration in water over the range of 0.5–10 mM, yielding best-fit regressions of the data that agree well with a data regression of microplate luminometer results. The data suggest that fluorescence can be used for the measurement of salivary sodium ion concentrations in low-resource or point-of-care settings. With further fluorescent assay testing, the device may find application in a variety of enzymatic or chemical assays.

## 1. Introduction

Saliva sampling via noninvasive qualitative and quantitative techniques is important in drug testing, as well as for monitoring physiological systems [1]. Saliva has a significant advantage over “classical” biological fluids, such as blood and urine, since it is more readily accessible and easily collected [2]. Because of the growing interest in minimally or noninvasive health assessments, the effectiveness of saliva as a means of determining patient health or diagnosing disease has grown in recent years, especially in low-resource or point-of-care settings [3]. While much attention is being paid to detect hormones or infectious diseases via rapid salivary testing, there has been less effort given to quantifying the levels of important, physiological indicators, such as the concentration of sodium ions. Quantitation of sodium ion levels in saliva can potentially be used to monitor patient wellness, track endogenous cycles, as well as to conduct screening for patients with chronic underlying diseases or those undergoing dialysis [4,5].

Saliva contains two major types of protein secretions: (1) a serous secretion that contains ptyalin (an α-amylase), which is an enzyme for digesting starches; and (2) mucus secretion that contains mucin for lubricating and for surface-protective purposes [6]. Saliva also contains especially large quantities of potassium, sodium and bicarbonate ions. Endogenous hormonal cycles in women and kidney function in dialysis patients can be tracked by quantifying the concentration of saliva from patient samples [7]. Of particular utility for this purpose are ion-specific fluorophores that can detect sodium ions in saliva, even in the presence of high levels of potassium ions [8]. The possibility of adding to a saliva sample a premixed fluorophore solution specific for sodium ions followed by measuring the fluorescence directly after mixing, without additional sample preparation procedures or chemical separation steps, would seem to be an attractive means for rapid analysis of this important indicator of physiological function. It should be noted that saliva sampling has specific protocols that yield different concentration levels of analytes depending on the way that the sample is collected [1,2]. Some protocols call for freezing prior to analysis in order to minimize the unwanted rheological properties of this complex fluid. These considerations, while noteworthy, may be less problematic if a method that features direct acquisition and analysis of saliva can be performed rapidly in low-resource settings. Moreover, the simplicity of data acquisition could motivate clinical researchers to use sodium ion concentrations from so-called “raw” saliva sample in studies and to relate those data to physiological changes.

Our primary objective in this experimental study was to determine if smartphones could be utilized for quantifying sodium ions in simple aqueous solutions at concentrations relevant to saliva monitoring. While these mobile devices are seemingly ubiquitous, there are a number of practical and technological hurdles that need to be overcome in order to realize their potential in health monitoring. It is beyond the scope of this paper to address the practical issues relating to the availability of smartphones for mixed use as a personal device and as a patient monitoring system, but it is the intent of this paper to offer a low-cost hardware design and image-processing method that can provide sensitivity and detection ranges similar to what is found with a laboratory microplate luminometer. Of particular concern is that some fluorophores require a high intensity of light and/or suitable optical filters in order to collect reliable fluorescence readings. Moreover, smartphone cameras are generally fitted with infrared filters, use a pixel array biased towards the green channel and have limited lens capabilities or settings. The design approach described in this paper is based on careful consideration of these and other limitations and should be regarded as one of many possible ways of addressing the technological challenges of using smartphones for quantitative fluorescent assays.

## 2. Experimental Section

### 2.1. Sodium Green Indicator Dye

The sodium ion indicator Sodium Green™ (Life Technologies Inc., Carlsbad, CA, USA) was chosen for this study, because it exhibits good selectivity for sodium over potassium ions, due to its ion size-specific crown ether cavity, and displays a much higher quantum yield in solutions containing Na^+^ [8]. Sodium Green is often used in biological research because of its low toxicity, and the tetramethylammonium form of the fluorophore does not penetrate cells. When it is present in an excess of sodium ions, the fluorescence reading can be used to determine the sodium ion concentration from aqueous and saliva solutions. The dissociation constant (K_d_) for Sodium Green for Na^+^ is 6 mM at 22 °C in potassium-free solution and above 21 mM at 22 °C in solutions containing both Na^+^ and K^+^ (with a total ion concentration of 135 mM) [8]. The K_d_ is dependent on pH, temperature (Sodium Green is stable at room temperature), ionic strength, levels of other cations and protein concentration. After binding to a sodium ion, there is an increase in fluorescence emission intensity of the Sodium Green with little shift in wavelength. Sodium Green has a peak excitation wavelength of 507 nm and an emission wavelength of 532 nm.

### 2.2. Test Solutions

For this study, Sodium Green Tetra (tetramethylammonium) Salt (Life Technologies Inc.) solutions were prepared using the protocol referenced by the manufacturer [9]. Briefly, a concentrated stock solution of Sodium Green in analytical-grade DMSO (Life Technologies Inc.) was refrigerated and protected from light. Test solutions were prepared by diluting the stock solution in DMSO (final concentration of 5 µM) and were found to remain viable in dark containers for at least 1 month at room temperature. Refrigeration was also used to store the diluted fluorophore solution, extending its suitability as a reagent for at least 4 months, but repeated thawing and freezing was found to significantly reduce this shelf life. Sodium Green Tetra Salt was added to solutions with known, but differing Na^+^ concentrations. Sodium ion solutions were prepared using DI water by serial dilution. Stock solutions of 0, 0.5, 1, 2, 5, 7, 10, 50 and 100 mM sodium ion were used to obtain calibration data to compare the smartphone system with the laboratory microplate luminometer.

Three images were taken for each Na^+^ concentration level for all smartphones and the luminometer. Additionally, a test solution to simulate human saliva, following the protocol for simulated saliva (SS1) was used [10] in order to determine the operability of the system and method for samples more closely resembling the properties of human saliva. A 1:1 ratio of sodium ion (290 µL) to Sodium Green solution (290 µL) was used for all smartphone measurements, while a 1:1 ratio at 100 µL each was used for the luminometer.

### 2.3. Sample Holder, Chamber and Smartphone Covers

**Figure 1:**
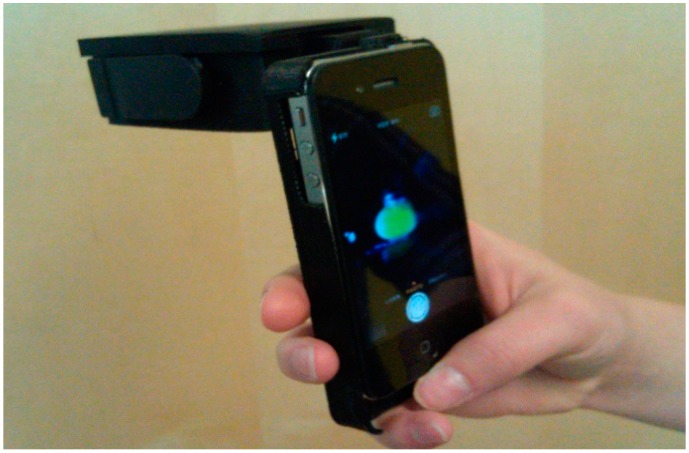
3D-printed housing with blue LED light (located inside housing) and detachable, 3D-printed iPhone 4 holder (shown without transmission grating). The system quickly connects different smartphone models to the housing, using an Archimedes screw design, while minimizing ambient light.

In order to excite the fluorophore and detect fluorescence intensity with a smartphone, a 3D-printed rectangular chamber with similarly 3D-printed covers for each phone model was used. The rectangular chamber consisted of a simple constant voltage circuit, two 3-Volt watch batteries in series and a 470-nm photodiode (Industrial fiber Optics, Tempe, AZ, USA). Please refer to Supplementary Material for Figures S1 and S2, which show 3D sketches that illustrate the optical system and smart phone adapter. This modular design required a detachable system, and an Archimedes screw design was used in order to connect the lens of the smart phone camera in the line-of-sight with the sample when placed in the sample chamber (Figure 1). Please refer to Figure S3 and S4, which show the Archimedes screw design. Using this design concept, we were able to build systems and acquire data with the following models: iPhone 4, iPhone 4s, iPhone 5, HTC Incredible I, Samsung Galaxy S3 and S4 and Nokia Lumina 920.

The transparent sample holders for the analysis and fluorophore solutions were 3D-printed oblate spheroids (iMaterialise) of a 590-microliter liquid capacity. This design concentrates the excitation source non-axially-symmetrically within the sample, generating a high intensity light as a cuspoid catastrophe within the chamber. A detailed analysis of the selection of this geometry and the concentrating effect is explained elsewhere [11]. Briefly, an optical caustic due to non-axially-0symmetric focusing of light creates regions of high intensity that greatly exceed the incident light intensity [11]. For fluorescence measurements using the design shown in Figure 1, an added benefit for image processing is that the emitted and reflected light are viewed by the smartphone camera in side-view, since the light is chromatically dispersed due to refraction from the sample chamber. Color separation into the fluorescent spectrum is thus simplified using this sample chamber geometry. An additional benefit of the lack of axial symmetry for the excitation light is that some of the light is trapped due to internal reflections and passes through the longitudinal axis several times [12,13] within the chamber until it is absorbed or generates fluorescence.

**Figure 2:**
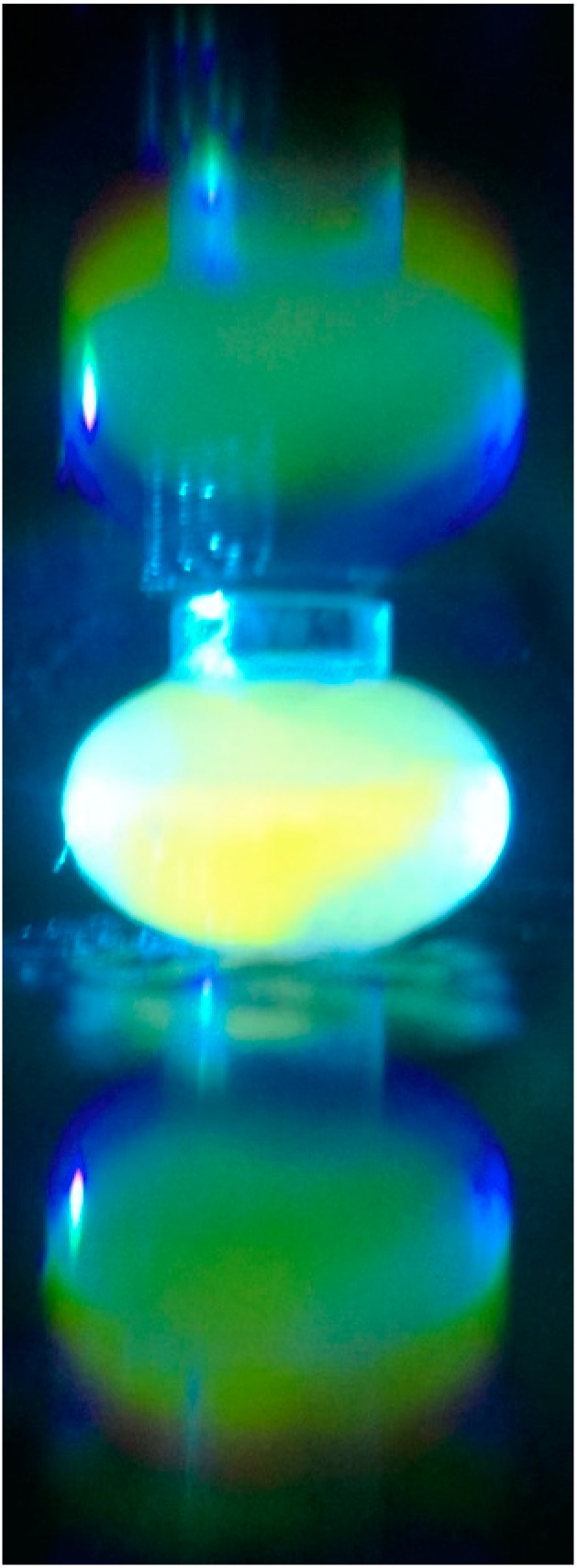
Image illustrating the effect of combining refractive chromatic dispersion with diffraction using a transmission grating in order to separate excitation and emission wavelengths prior to digital image processing.

Another design feature simplifying measurement without adjusting camera settings or specifying a particular model or manufacturer of smartphones is the use of a holographic grating placed in front of the camera lens. A 1000 lines/mm transmission holographic diffraction grating film (Scientific Equipment of Houston, Navasota, TX, USA) is used to separate the observed light into 1 direct and 2 virtual images, thereby minimizing photosensor cell saturation and further separating the wavelengths observed through diffraction (Figure 2). As a further indication of the utility of this non-standard optical device, a spectral image of the fluorescence and excitation light was obtained using a 3D-printed slit, a holographic diffraction transmission grating and a longer “barrel” with a 17-degree tilt. The results from this smartphone spectrometer were compared to the fluorophore distributor’s emission spectra published on the Internet [8]. Calibration of the smartphone spectrometer followed a standard procedure for low-resolution calibration [14], namely matching pixel distance with spectral lines from laboratory fluorescent lamps, using only a linear scaling factor to match pixel distances to emission line wavelengths.

### 2.4. Laboratory Microplate Fluorescence Instrument

In order to measure sodium ion concentration, a Lonza Model Orion II Microplate Luminometer was operated using 488-nm excitation light and 520-nm or 590-nm emission filters. In order to mimic the procedures and solution concentrations used with the smartphone device, 100 microliters of sodium ion solution and 100 microliters of Sodium Green fluorophore solution were placed in triplicate wells. Reference wells containing 200 microliters of DI water were used as a blank. All measurements were based on standard software settings, as recommended by the manufacturer, for fluorescence.

### 2.5. Image Processing

All images were analyzed using ImageJ (NIH, http://imagej.nih.gov/ij/). RGB images were separated into three color channels, red (R), green (G) and (B) blue, using the built-in IMAGE > COLOR > SPLIT CHANNELS function. Once the color channels are separated, either an area in the center of the sample chamber or an area of one of the two virtual images was selected using a circle area tool. For the initial set of measurements using the true sample chamber image, reflection spots at the light entrance or light exit were excluded. Using the EDIT function in ImageJ, the selection manager was used in order to acquire the same area for all images. The ANALYZE menu of ImageJ was then employed to obtain integrated density, mean pixel intensity and pixel area values for the selections.

### 2.6. Computational

Wolfram Mathematica 10.0 software (Wolfram Research, Oxfordshire, UK, 2013) was used for graphing approximate solutions for the 3D intensity functions along with numerical integrations to scale results for the spherical and oblate spheroid sample chambers.

## 3. Results and Discussion

### 3.1. Sodium Ion Detection Using Fluorescence

For a crown ether-linked fluorophore Sodium Green Tetra, present in an excess of sodium ions, free sodium ion concentration in solution can be calculated using the following equation [15]:

(1) $${\left[Na^{+}\right]}_{free}\;=\;K_{d}\left[\frac{F-F_{min}}{F_{max}-F}\right]$$

where *F* is the relative fluorescence reading, *F_min_* is the relative fluorescence reading in the absence of sodium and *F_max_* is the relative fluorescence of the sodium ion-saturated fluorophore. The equilibrium dissociation constant is a molecular property of Sodium Green Tetra that is dependent on the solution conditions, but independent of the instrument used to quantify fluorescence. For the quantitation range of interest, the total sodium ion concentration is much greater than the fluorophore concentration, thus allowing for the assumption that the free sodium ion concentration is equal to the initial sodium ion concentration. Based on this assumption and by rearranging Equation (1), the experimental data can be linearized by plotting the initial sodium ion concentration and the fluorescence as per Equation (2):

(2) $$\frac{{\left[Na^{+}\right]}_{initial}}{F-F_{min}}=\frac{{\left[Na^{+}\right]}_{initial}}{F_{max}}+\frac{K_{d}}{F_{max}}$$

It should be noted that linearizing the equation in this fashion biases curve fitting to data points in the middle and high concentration range, which is appropriate, since *K_d_* is determined to be approximately 6.7 mM, and this agrees well with the manufacturer’s value for potassium-free solutions.

**Figure 3:**
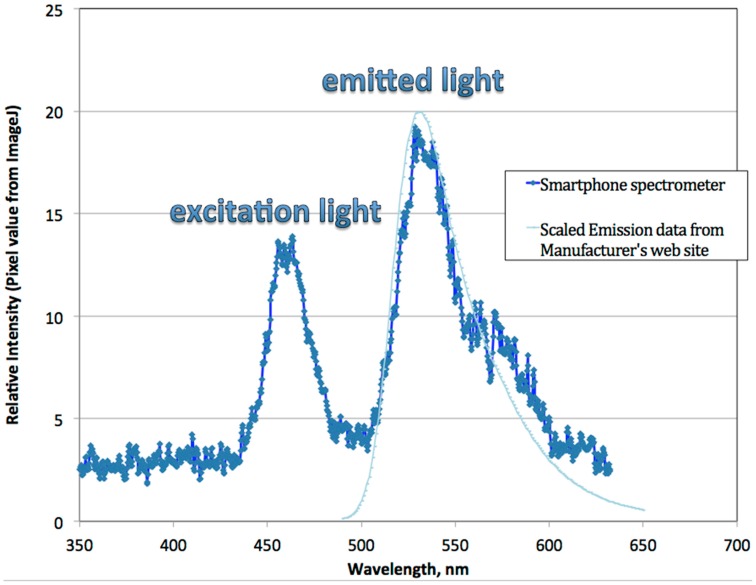
Smartphone fluorimeter spectra resolving the excitation and emitted light and compared to graphed data from the distributor of Sodium Green Tetra [8].

**Figure 4:**
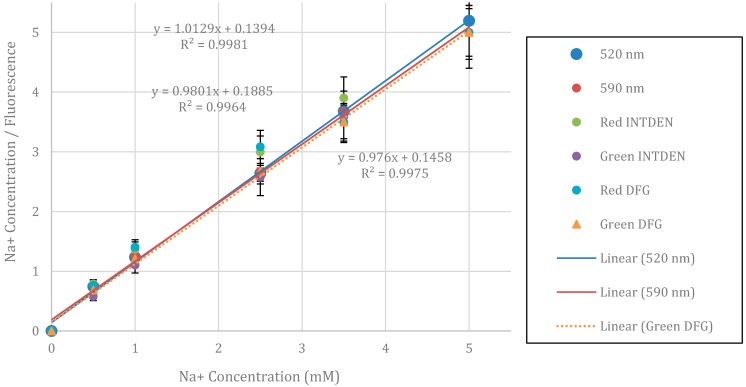
Linearized plot of fluorescence with a 520-nm (top equation and R^2^ value) and 590-nm emission filter (equations to the left of the lines and R^2^ value are measured using the manufacturer’s supplied software, FluoroPLUS, TiterTek Instruments, Huntsville, AL, 2009). The other data represent the red and green integrated intensity values as measured using the integrated density of the red image channel (RED INTDEN) and the same area for the green image channel (GREEN INTDEN). Data where a holographic grating is used in front of the camera lens are designated as Red DFG and Green DFG respectively. The dotted trend line and lower-right equation are for the Green DFG, which had the best correlation when compared to the microplate reader, especially at the lower sodium ion concentrations. Relative fluorescence readings are determined based on normalizing with respect to the fluorescence measured at a 10 mM initial sodium ion concentration for each instrument. Simulated saliva (SS1) is used for the data at a 5 mM Na ion concentration.

**Figure 5:**
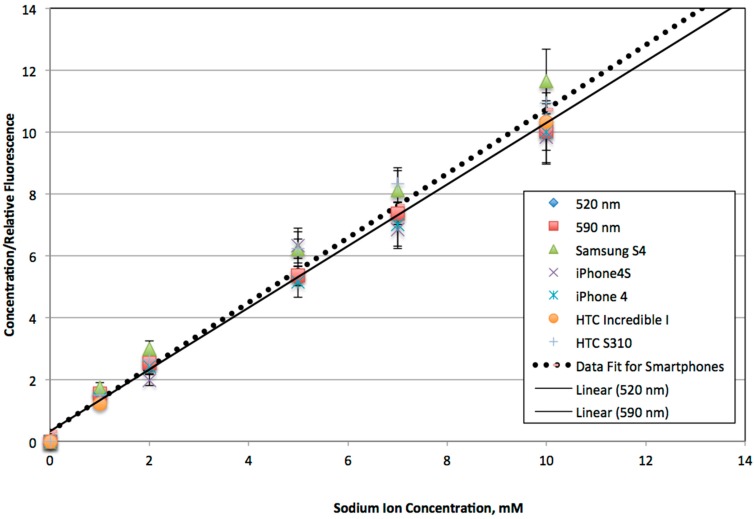
Another comparison series of the luminometer and smartphone fluorimeter as a function of initial sodium ion concentration along with the best-fit linear correlations are shown. All smartphones were equipped with a holographic grating, and the green channel was used to calculate fluorescence based on integrated density measurements. Relative fluorescence readings are used based on normalizing with respect to the fluorescence measured at a 10 mM initial sodium ion concentration.

Figure 3 is a comparison of the emission spectra from the fluorophore distributor’s website [8] with data using the smartphone spectrometer. After scaling the relative peak heights, this figure shows that the measured emission maximum shows very good agreement with the expected value. However, there is a shoulder between 560 and 570 nm in the measured data that is not accounted for in the published data. Comparisons of fluorescence using a laboratory microplate fluorimeter with 520- and 590-nm emission filters and green and red digital filters for the smartphone system are used in Figure 4 and Figure 5 to investigate whether this discrepancy is meaningful for sodium ion quantitation.

In Figure 4, initial testing of the red and green fluorescence values for an iPhone 4 are shown with and without a holographic diffraction film and compared to laboratory microplate fluorescence readings with emission filters at 520 and 590 nm. Linear regressions for both emission filters compare very well with the regression line using the green fluorescence data and a holographic film for the iPhone 4. Based on these results, further comparisons of microplate data with a variety of other smartphones using a diffraction grating and the green integrated density measurements are plotted in Figure 5.

The following section discusses in more detail how the refraction method, the sample chamber and the device design accessories contribute to the capabilities of the smartphone fluorimeter.

### 3.2. Discussion

#### 3.2.1. Utility of Diffraction Grating for Avoiding Pixel Saturation

A typical smartphone camera lens focuses light onto a sensor consisting of light-sensitive photosites, which are usually square and arranged in a grid pattern. It is of particular importance to note that the area of the photosite affects the signal-to-noise ratio, and smartphones contain some of the smallest camera sensor photosites found in commercial digital cameras. For example, the iPhone 4 has photosites of 1.75 microns, which yields a resolution of 2592 × 1936 pixels or 5.0 megapixels. Each photosite is usually covered with an array of RGB filters in a Bayer pattern, with odd-numbered rows containing alternating red-green filters and even-numbered rows alternating green-blue filters. The Bayer pattern results in twice as many green- as red- or blue-sensitive cells, leading to an increased sensitivity to luminance in the green portion of the visible spectra.

**Figure 6:**
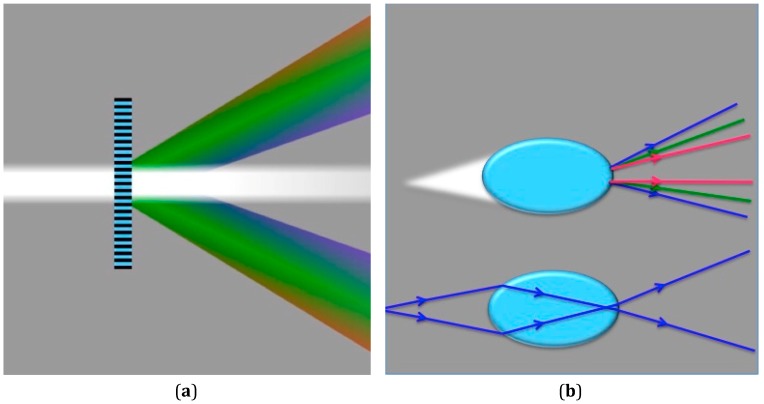
(**a**) The color separation of white light through a diffraction grating film. (**b**) (Top) Chromatic dispersion for a white light point source emitting from a focal zone inside the oblate spheroid due to refraction (color separation exaggerate for clarity) and (bottom) two rays refracting from a blue light source to help clarify that there is a focal zone within the sample chamber. The image in (**a**) was adapted from Cmglee (GFDL (http://www.gnu.org/copyleft/fdl.html)), via Wikimedia Commons, in order to provide a visual reference to compare with the chromatic dispersion effect shown in (**b**).

Although an iPhone 4 has a resolution of 2592 × 1936 pixels, the spectral band of the sensor covers approximately 750 × 100 pixels in the dispersive direction [16]. With a minimum focal length of 3.85 mm for the iPhone 4, the wavelength separation between adjacent pixels in the spectral direction is only 0.333 nm·pixel^−1^ [16]. The smartphone fluorimeter holds the sample chamber at 4.5 mm from the camera lens, so that it is not too close to the lens, and hence, the chamber can be in focus for image collection. However, when a 1000-lines per mm holographic grating film is placed directly in front of the smartphone lens’ glass cover, the light is diffracted, so that the image is recorded by the CCD element as two virtual images of lower intensity with one true image in the middle (see Figure 2 and Figure 6). The true image or one of the virtual images can be analyzed after digital color processing, post-acquisition. The utility of this geometric configuration when imaging the “true” image is that it mitigates oversaturation in nearest neighbor photosites, without modifying the smartphone or necessitating specific software to lower overall image brightness. Since the fluorescence measurement depends on detecting the light from the green portion of the visible spectrum, this diffraction effect is important for Bayer-patterned photosites, because the pixel values in the image are well within the range of quantitation, especially after separating the green channel digitally. However, there is a further advantage of using a transmission grating that can be realized by quantifying the integrated density from a virtual image, and this is explained in the next section.

#### 3.2.2. Chromatic Dispersion and Aberrations

Usually, dispersion and aberrations are unwelcomed effects that are corrected and compensated for using lenses and planar sample chamber geometry. However, the need for wavelength separation and higher photon sensitivity when measuring fluorescence can be at least partially met using geometries specifically designed to create these effects. As seen in Figure 6b, the oblate spheroid sample chamber disperses the blue excitation more than the green and red emitted light due to refraction, since the indices in water for 470, 520 and 600 nm are 1.339, 1.337 and 1.334, respectively. Based on the red and blue refractive indices and the focal distance, 470- and 600-nm light originating from the sample chamber is separated by about seven pixels, assuming that the diffraction grating was not placed in front of the camera lens cover. However, with the diffraction grating, there is a further separation of the colors in the two virtual images, since longer wavelengths of light are diffracted more than shorter wavelengths (Figure 1 and Figure 6). Thus, as noted in the data provided in Figure 3, at a low sodium ion concentration, the combined methods of maximizing wavelength separation to the sensor improves detection linearity due to more accurate digital color filtering and the acquisition of a higher level of total light captured. Moreover, the optical path length from the sample to the camera can be kept near the minimum focal length, which results in higher intensities of light collected by the camera’s sensor.

An added benefit of using a spheroidal sample chamber is that the excitation light is refracted to form a 3D focal zone, without the use of an external lens. It is advantageous to intensify the light within the sample chamber, since fluorescence intensity is proportional to the excitation light intensity. Previously, we illustrated how the higher intensity or “focal zones” can be found in the interior of an oblate spheroid, whereas spherical lenses of a uniform refractive index have a focal zone just outside the lens [11]. While refraction of light from a planar source into an oblate spheroid sample chamber leads to a focal zone (for *p* = 1 rays) with relatively high intensity due to constructive interference [17], it also allows some excitation light to reflect and concentrate near the major axis of the projected ellipse [12,18] (for *p* = 2 and *p* = 3 rays), until it is absorbed by the fluorophore, leading to fluorescence emission (depending on its quantum efficiency). These “excitation light-shaping effects” are explained in the paragraphs below by discussing thick lens effects, using spherical and axicon lenses as references.

**Figure 7:**
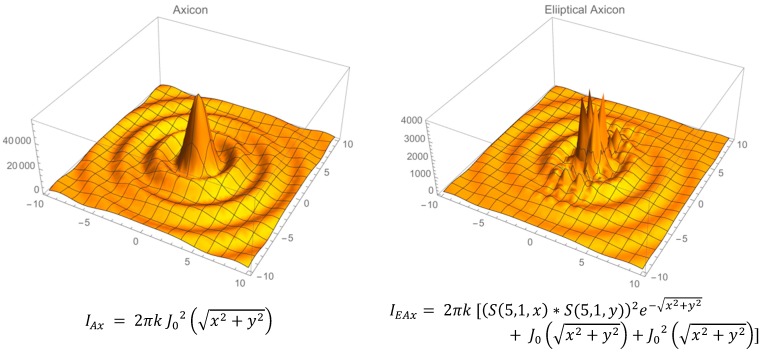
Equations to simulate intensity profiles for regular and elliptical axicon intensity profiles at a particular axial distance Z ≤ focal length [23,24]. The function S is the Mathieu sine function, and k is the angular wavenumber.

Among the options for “thick lens” focusing of light, spherical lenses have been studied for enhancing the intensity of light in solar energy [17], and axicon lenses have been used to transform a Bessel beam into a uniform ring of light that is self-focusing, for eye surgery applications [19]. Axicon lens near-field intensity models and experimental data are well described in the literature. Figure 7 gives approximate equations with accompanying 3D intensity plots for a regular and an elliptical axicon based on the approach outlined by Berry and Howls [20] and more detailed models and data from several sources [21,22]. The transition from a Bessel beam with concentric rings of equal intensity for a regular axicon to a cluster of high-intensity peaks in an elliptical axicon [23] illustrates how loss of axial symmetry generates optical caustic zones due to constructive interference. Thaning and colleagues refer to this zone as having the overall shape of an “astroid” [21].

The changes noted in the intensity patterns in the elliptical axicon were very similar to the pattern changes noted by Nye and Marston for light scattering (e.g., far field) from a regular *vs.* an oblate sphere [9,25,26,27]. They referred to the caustic zone pattern as a “HUFS” or “hyperbolic umbilic focal section”. The “astroid” shape discussed by Thaning and colleagues and the “HUFS” shape described by Nye and Marston suggest that a similar constructive interference zone transition occurs in the near field when a spheroid is distorted along one axis to form an oblate spheroid.

**Figure 8:**
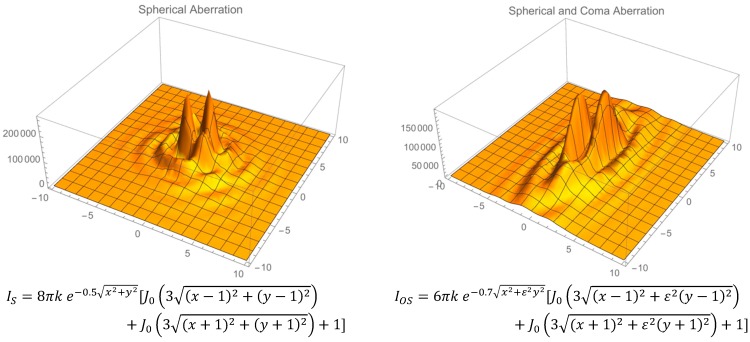
The intensity profiles are plotted using equations that simulate the spherical and spherical with coma aberrations at the optical caustic focal length [20,30]. In the right panel, the parameter ɛ is the eccentricity of the oblate spheroid, and as in Figure 4, k is the angular wavenumber.

Figure 8 follows the format in Figure 7, showing approximate equations for the intensity in a sphere and oblate spheroid, respectively, for planar illumination of light. These equations are based on data and detailed near-field mathematical models [17,28], as well as, in part, on expected forms of the Fresnel–Kirchhoff near-field integral solutions due to spherical and coma aberrations [23,29].

**Figure 9:**
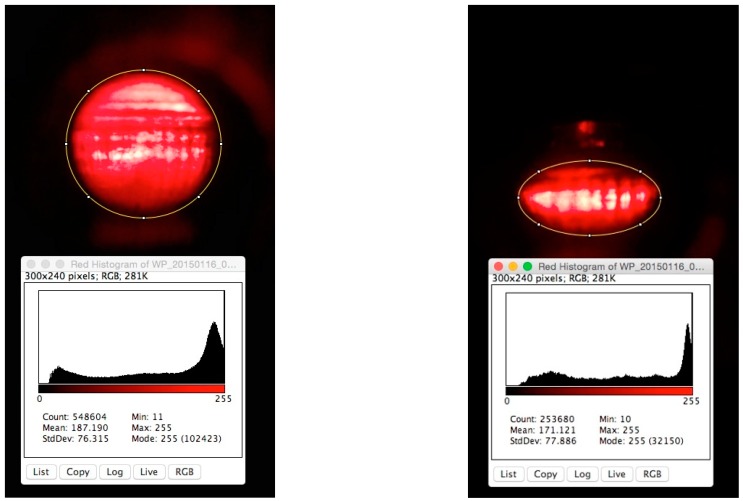

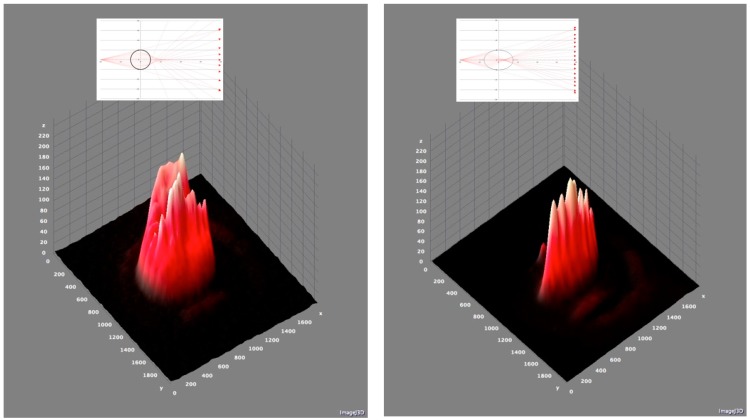
Images of a spherical and an oblate spheroid sample chamber using a red light (620 nm) source in direct line with the camera sensor. The sample chambers are filled with water. The top panels show the images and a histogram of the pixel intensities for the RGB images. 3D renderings of the data and inserts showing 2D ray tracings are given in the lower panels for qualitative comparisons to the graphs in Figure 8.

In order to compare the relative intensities shown in Figure 8, it is assumed that the total power is the same, which requires that the integral of each equation with respect to the area shown are matched. This was accomplished by adjusting two parameters: namely, the constant multiplied by the angular wavenumber and the fraction in the exponential term. As expected, the peak intensities for the spherical lens are higher, while there are two distinct regions of peak intensities in the oblate spheroid that are offset due to coma aberration. Based on this prediction, a series of images using spherical and oblate spherical sample chambers was collected using a lower intensity red LED in direct line with the smartphone camera. Figure 9 illustrates a typical image taken (for each sample chamber geometry) along with a corresponding pixel intensity contour plot.

In a manner similar to geometric considerations and predictions using the equations in Figure 8, in Figure 9, the oblate spheroid chamber image shows a band of high intensity along the equator of the major axis. The images taken by the camera sensor in Figure 9 are beyond the focal zone of each sample chamber, and hence, there is a broadening of the intensity profile. Furthermore, due to pixel saturation, these images should only be considered for qualitative comparison of the high-intensity zones for both sample chambers. These images along with the equations in Figure 8 indicate that for fluorescence measurement (e.g., excitation light illuminates the sample chamber in a position orthogonal to the camera sensor in that case), there will be a wide zone of high-excitation light intensity in the center of the oblate spheroid sample chamber. Based on 2D ray tracing inserts [11] given in Figure 9 for comparison purposes, it is seen that this zone reaches a higher intensity inside the sample chamber than for a spherical sample chamber, and thus, detection is more sensitive at lower analyte concentrations for an oblate spheroid sample chamber.

## 4. Conclusions

Refraction and diffraction were used in a new device coupled to a variety of smartphone models in order to track sodium ion aqueous solutions from 0–10 mM concentrations using fluorescence. The crown ether fluorophore protocol was also performed with a laboratory microplate luminometer, and the data regression lines for both instruments showed very good agreement. The unique oblate spheroid sample chamber produces a non-axially symmetric focal zone that concentrates light within the sample and, based on the literature, oscillates a fraction of the excitation due to internal reflection. Ray tracing and mathematical models are used to illustrate the combined utility of the device design. It is anticipated that saliva testing or other biological and chemical assays that rely on fluorescence could be performed on the handheld device, thus amplifying the utility of these assays for field and/or low-resource settings.

## Supplementary Files

Supplementary File 1

## References

[1] Marques M., Loebenberg R., Almukainzi M. (2011). Simulated biological fluids with possible application in dissolution testing. Dissolution Technol..

[2] Life Technologies, Sodium Green™ Indicator in The Molecular Probes Handbook. http://www.lifetechnologies.com/us/en/home/references/molecular-probes-the-handbook/indicators-for-na-k-cl-and-miscellaneous-ions/fluorescent-na-and-k-indicators.html.

[3] Kaushik A., Vasudev A., Arya S.K., Pasha S.K., Bhansali S. (2014). Recent advances in cortisol sensing technologies for point-of-care application. Biosens. Bioelectron..

[4] Motamayel Ahmadi F., davoodi P., Dalband M., Hendi S.S. (2010). Saliva as a mirror of the body health. DJH.

[5] Pels E. (2013). Oral hygiene status and selected saliva biomarkers in children with acute *lymphoblastic leukaemia* during anticancer therapy. J. Leuk..

[6] Hall J.E. (2011). Guyton and Hall Textbook of Medical Physiology.

[7] MathWorks Converting Color Data between Color Spaces. http://www.mathworks.com/help/images/converting-color-data-between-color-spaces.html.

[8] Malon R.S.P., Sadir S., Balakrisdhnan M., Corcoles E.P. (2014). Saliva-based biosensors: Noninvasive monitoring tool for clinical diagnostics. BioMed Res. Int..

[9] Blicharz T., Rissin D., Bowden M., Hayman R., DiCesare C., Bhatia J., Grand-Pierre N., Siqueira W.L., Helmerhorst E.J., Loscalzo J. (2008). Use of colorimetric test strips for monitoring the effect of hemodialysis on salivary nitrite and uric acid in patients with end-stage renal disease: A proof of principle. Clin. Chem..

[10] Marston P.L., Trinh E.H. (1984). Hyperbolic umbilic diffraction catastrophe and rainbow scattering from spheroidal drops. Nature.

[11] García A.A., Nuñez L., John C., Hadish H., Orioke V., Mujica V. (2013). Application of Newton’s zero order caustic for analysis and measurement: Part-I absorbance. Int. Res. J. Pure Appl. Chem..

[12] Yang M., Wu Y., Sheng X., Fang Ren K. (2015). Comparison of scattering diagrams of large non-spherical particles calculated by VCRM and MLFMA. J. Quant. Spectrosc. Radiat. Transf..

[13] Yu H. Laser Beam Interaction with Spheroidal Droplets: Computation and Measurement. http://tuprints.ulb.tu-darmstadt.de/3714/7/Dissertation.pdf.

[14] Public Lab Spectrometer. http://publiclab.org/wiki/spectrometer.

[15] Szymacinski H., Lakowicz J.R. (1997). Sodium green as a potential probe for intracellular sodium imaging based on fluorescence lifetime. Anal. Biochem..

[16] Gallegos D., Kenneth L., Yu H., Clark P., Lin Y., George S. (2013). Label-Free biodetection using a smartphone. Lab Chip.

[17] Kofler J., Arnold N. (2006). Axially symmetric focusing as a cuspoid diffraction catatastrophe: Scalar and vector cases and comparison with the theory of Mie. Phys. Rev. B.

[18] Sosa-Martinez H., Gutierrez-Vega J.C. (2009). Optical forces on a Mie spheroidal particles aribitrarily oriented in a counterpropagating trap. J. Opt. Soc. Am. B.

[19] Palima D., Gluckstad J. (2013). Gearing up for optical microrobotics: Micromanipulation and actuation of synthetic microstructures for optical forces. Laser Photonics Rev..

[20] Berry M.V., Howls C.J. (2010). Axial and focal-plane diffraction catastrophe integrals. J. Phys. A Math. Theor..

[21] Thaning A., Friberg A.T., Popov S.Y., Jaroszeqicz Z. (2002). Design of diffractive axicons producing uniform line images in Gaussian Schell-model illumination. J. Opt. Soc. Am. A Opt. Image. Sci. Vis..

[22] Graf T., Moloney J., Venkataramani S. (2013). Asymptotic analysis of weakly nonlinear Bessel-Gauss beams. Phys. D: Nonlinear Phenom..

[23] Thaning A., Jaroszeqicz Z., Friberg A.T. (2003). Diffraction axicons in oblique illuminations: Analysis and experiments and comparison with elliptical axicons. Appl. Opt..

[24] Duocastella M., Arnold C.B. (2012). Bessel and annular beams for materials processing. Laser Photonics.

[25] Marston P.L., Kaduchak G. (1994). Generalized rainbows and unfolded glories of oblate drops: Organization for multiple internal reflections and extensions of cusps into Alexander’s dark band. Appl. Opt..

[26] Marston P.L. (1999). Catastrophe optics of spheroidal drops and generalized rainbows. J. Quant. Spectrosc. Radiat. Transf..

[27] Masmali A.M., Purslow C., Murphy P.J. (2014). The tear ferning test: A simple clinical technique to evaluate the ocular tear film. Clin. Exp. Optom..

[28] Kofler J. (2004). Focusing of light in axially symmetric systems within the wave optics approximation. Graduate Engineering Degree Thesis.

[29] Stamnes J.J., Hilger A. (1986). Waves in Focal Regions.

[30] Thompson K.P. (2010). Multinodal fifth-order optical aberrations of optical systems without rotational symmetry: The comatic aberrations. J. Opt. Soc. Am. A.

