# Supplementary material for: Handheld Device Adapted to Smartphone Cameras for the Measurement of Sodium Ion Concentrations at Saliva-Relevant Levels via Fluorescence
Source: Bioengineering (Basel). 2015 Jun 2;2(2):122–38. doi: 10.3390/bioengineering2020122 (PMC5597196; doi:10.3390/bioengineering2020122)
Supplement: Supplementary File 1 [file bioengineering-02-00122-s001.pdf]

Supplementary Information

Handheld Device Adapted to Smartphone Cameras for the Measurement of Sodium Ion Concentrations at Saliva-Relevant Levels via Fluorescence

---

The following low-resolution, 3D sketches are provided to illustrate the optical system and smartphone adaptor used to generate data using the handheld device. All pieces are shown in white for clarity of presentation, but they were in fact 3D printed using black filament.

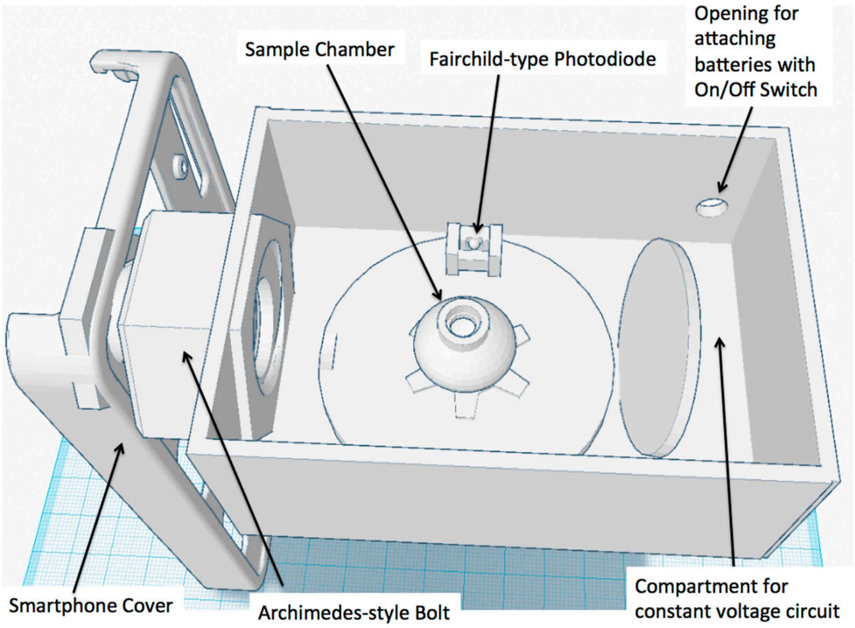

Figure S1. Overall, low-resolution, 3D schematic of the smartphone system. Key features are labeled, but the cover is not shown in this schematic.

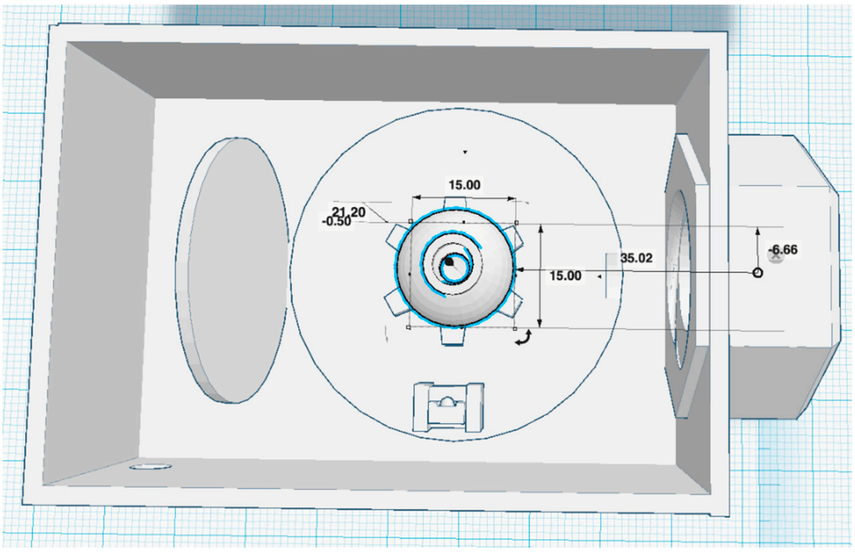

Figure S2. Sample chamber dimensions and distance from the camera lens are provided in this sketch. All measurements are in millimeters.

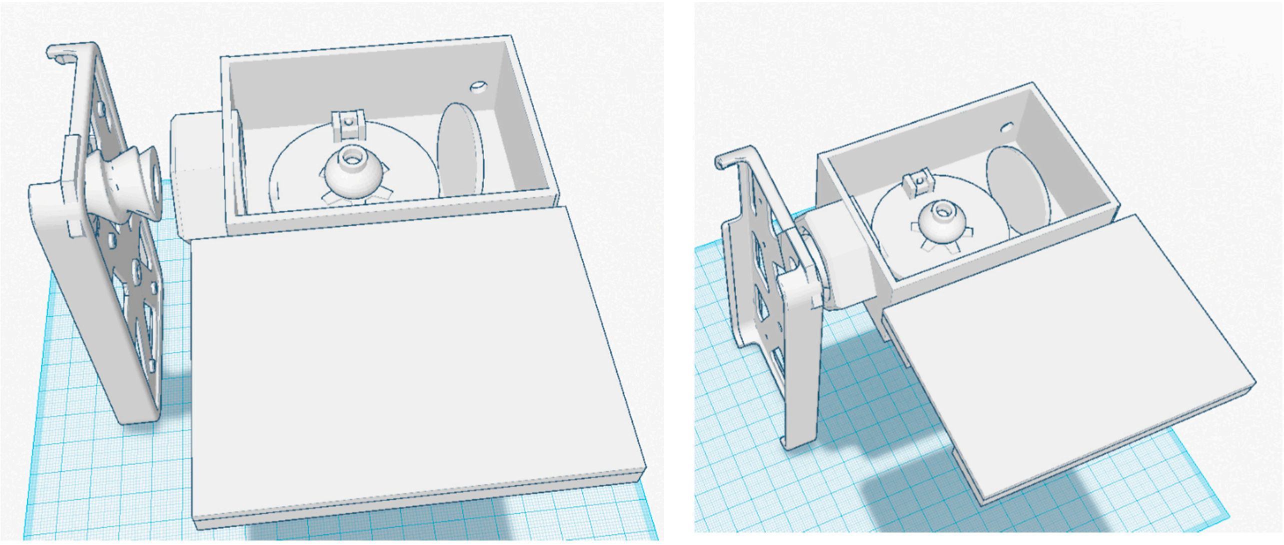

(a) (b)

Figure S3. View of the Archimedes screw on the smartphone holder (**a**) before attaching to the excitation light base and (**b**) attached to the base by rotating the screw or base.

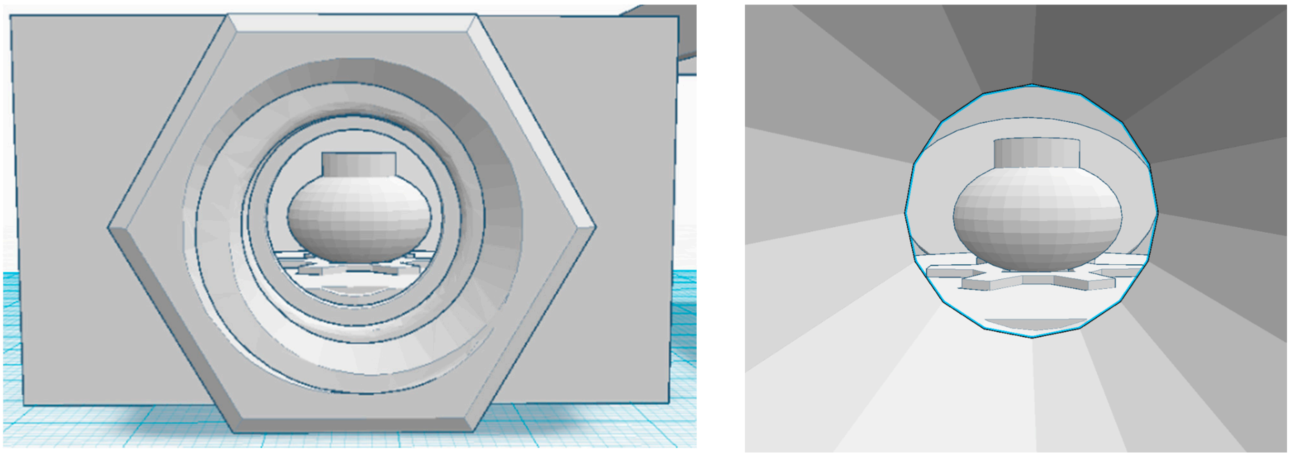

(a) (b)

Figure S4. View through the opening of the base: (**a**) without the smartphone holder; and (**b**) the field of view through the smartphone with the holder attached to the base, as seen in Figure S3b.

© 2015 by the authors; licensee MDPI, Basel, Switzerland. This article is an open access article distributed under the terms and conditions of the Creative Commons Attribution license (http://creativecommons.org/licenses/by/4.0/).
